# Low kV Computed Tomography of Parenchymal Abdominal Organs—A Systematic Animal Study of Different Contrast Media Injection Protocols

**DOI:** 10.3390/tomography7040069

**Published:** 2021-11-29

**Authors:** Daniel Overhoff, Gregor Jost, Michael McDermott, Olaf Weber, Hubertus Pietsch, Stefan O. Schoenberg, Ulrike Attenberger

**Affiliations:** 1 Department of Radiology and Nuclear Medicine, University Medical Center Mannheim, Heidelberg University, 68167 Mannheim, Germany; stefan.schoenberg@umm.de; 2 MR and CT Contrast Media Research, Bayer AG, 13353 Berlin, Germany; gregor.jost@bayer.com (G.J.); hubertus.pietsch@bayer.com (H.P.); 3 Radiology Research and Development, Bayer AG, 13353 Berlin, Germany; michael.mcdermott1@bayer.com (M.M.); olaf.weber@bayer.com (O.W.); 4 Department of Diagnostic and Interventional Radiology, University Hospital Bonn, Rheinische-Friedrich-Wilhelms-Universität Bonn, 53127 Bonn, Germany; Ulrike.Attenberger@ukbonn.de

**Keywords:** contrast media, angiography, computed tomography, iodine delivery rate, low kilovolt, abdominal imaging

## Abstract

Objectives: To evaluate multiphase low kV computed tomography (CT) imaging of the abdomen with reduced contrast media (CM) dose using different injection protocols. Methods: Two injection protocols were evaluated for use with low kV (80 kV) multiphase abdominal imaging in comparison to the standard procedure acquired at 120 kV (500 mgI/kg; 5 mL/s). This evaluation was conducted in a highly standardized animal study (5 Goettingen minipigs). The low kV protocols consisted of (a) a single-flow (SF) injection with 40% reduced CM dose and injection rate (300 mgI/kg; 3 mL/s) and (b) a DualFlow (DF) injection protocol consisting of 60%/40% contrast to saline ratio administered at 5 mL/s. Dynamic CT was first performed within representative liver regions to determine optimal contrast phases, followed by evaluation of the three protocols in multiphase abdominal CT imaging. The evaluation criteria included contrast enhancement (CE) of abdominal organs and vasculature. Results: The 80 kV DF injection protocol showed similar CE of the abdominal parenchymatous organs and vessels to the 120 kV reference and the 80 kV SF protocol. Hepatic parenchyma showed comparable CT values for all contrast phases. In particular, in the portal venous parenchymal phase, the 80 kV DF protocol demonstrated higher hepatic parenchymal enhancement; however, results were statistically non-significant. Similarly, CE of the kidney, pancreas, and abdominal arterial/venous vessels showed no significant differences between injection protocols. Conclusions: Adapted SF and DF injection protocols with reduced IDR/iodine load offer the potential to calibrate optimal CM doses to the tube voltage in abdominal multiphase low kV CT imaging. The data suggest that the DF approach allows the use of predefined injection protocols and adaption of the contrast to saline ratio to an individualized kV setting and yields the potential for patient-individualized CM adaption.

## 1. Introduction

Low kV imaging in CT offers the possibility of both a significant reduction in radiation dose and a significant reduction in the amount of contrast medium (CM) to be administered. The opportunities of low kV imaging in CT angiography (CTA) have been well-studied by several investigators [1,2]. Due to the specific absorption properties of iodine, with increasing attenuation at lower kV levels, the possibility of up to a 50% reduction in CM dose in CTA at 70 kV has been shown [3,4]. This can be combined with low radiation dose scan protocols [5]. However, the relation between the reduction in CM dose, radiation dose, and image quality are less well-studied in the abdominal CT of parenchymatous organs. Initial clinical studies have investigated hepatic pathologies such as HCC in low kV imaging in CT mostly in Asian populations with lower body weight [6,7,8]. Recent technological innovations such as increased X-ray tube power and improved reconstruction techniques may allow for low kV abdominal CT to be utilized in an increasing patient population (6).

For parenchymal enhancement, the total iodine dose rather than the iodine delivery rate (IDR) is the most relevant parameter. The target enhancement for the healthy liver during portal venous phase is 50 HU, requiring an iodine dose of approximately 520 mgI/kg body weight at 120 kV [9]. Considering the CM dose reductions obtained in low kV CTA, a dose of 300 mgI/kg body weight should be sufficient to obtain a hepatic enhancement of 50 HU at 80 kV [4]. This can be implemented by utilizing individually adapted CM injection protocols [10]. The injection protocol for the 40% lower CM dose can be realized by simply keeping the injection duration constant and adapting the dose by reducing the injection rate and CM volume equally. This would result in a reduction in dose from 500 mgI/kg (120 kV) to 300 mgI/kg (80 kV) in our study, along with a reduced injection rate from 5 mL/s to 3 mL/s. An alternative approach is to simultaneously inject a contrast–saline mixture (DualFlow, DF). Here, the injection duration and injection rate are held constant, while the percentages of contrast and saline are adjusted according to the reduction factor. In our study, the DF mixture would be 60% contrast and 40% saline. With the first approach, reductions in injection rate on the order of 40% may alter the bolus timing and kinetics of CM distribution [11,12], which could result in suboptimal image delay times for multiple contrast phases. The DF injection protocols may offer an alternative approach, mitigating the potential effects of reducing the injection rate.

The aim of this highly standardized animal study was to evaluate these two types of individually adapted injection protocols for low kV (80 kV) multiphase abdominal imaging in reference to the standard injection protocol and procedure acquired at 120 kV.

## 2. Materials and Methods

In the first part of the study, the contrast enhancement (CE) for the three protocols was monitored by dynamic CT imaging of a small part of the liver centered at the level of the division of the portal vein (imaging without table feed). Based on the resulting time-attenuation curves (average values obtained from all subjects), optimal acquisition times of the different clinically relevant CE phases (unenhanced, early arterial, late arterial, portal venous vascular, portal venous parenchymal, nephrographic, late phase) were defined. In the second part, multiphase CT examinations of the entire abdomen were performed for each of the three protocols. The evaluation criteria included CE of the different abdominal organs as well as the vascular target structures for the optimized CE phases.

### 2.1. Animal Model

All animals (five female Goettingen minipigs) were handled with the approval of the State Animal Welfare Committee and in compliance with the German Animal Welfare Legislation. The studies were performed under general anesthesia as described in the literature [13].

Animals were placed in a prone position and CT imaging was performed on two different days. Initially, the animals underwent dynamic CT with small spatial coverage. One week later, the three protocols were compared in multiphase abdominal CT imaging. This was performed during end-expiratory ventilation stop. The heart rate was monitored prior to the CM injection. The order of the three protocols was randomized for each animal for both study parts. A resting time of at least 45 min was allowed between examinations to minimize residual enhancement of the parenchymal organs and blood pool.

### 2.2. CT Imaging

All images were acquired with a third-generation dual-source CT scanner (Somatom Definition Force, Siemens Healthineers, Forchheim, Germany).

The CT scan parameters of part 1 and part 2 of this study are summarized in Table 1.

For part 1 of the study, a dynamic CT without table feed was performed for each protocol at the level of the liver and portal vein for 105 s, with a gap between the scans of 1.5 s and simultaneous start of CT scan and CM injection.

For part 2 of the study, a CT of the entire abdomen was performed for each protocol and each optimized contrast phase. After a non-enhanced scan, bolus tracking on the thoracic descending aorta with a threshold of 100 HU was used as a trigger.

Subsequently, the following phases were acquired using fixed scan delays determined in part 1: early arterial (5 s), late arterial (11 s), portal venous vascular (40 s), portal venous parenchymal (69 s), nephrogenic (80 s), late (180 s).

### 2.3. Injection Protocols

Iopromide 300 mgI/mL (Ultravist 300, Bayer Vital GmbH, Leverkusen, Germany) and an injection system with an approved DualFlow option (MEDRAD^®^ Centargo, Bayer U.S. LLC, Indianola, PA, USA) were used for all injection protocols. The iodine dose was adapted by body weight using a dosing factor of 500 mgI/kg for 120 kV SF, and a 40% reduced iodine dose of 300 mgI/kg for 80 kV protocols. The CM was preheated to 37 °C and injected according to the protocols summarized in Table 2.

### 2.4. Fluid Delivery Measurements

Additionally, the dynamics of the different CM injection protocols were assessed over time using a MicroMotion 5700 Coriolis Transmitter (Emerson Electric Co., St. Louis, MO, USA). The transmitter was positioned at the outlet of the catheter to track the iodine delivery rate (in grams per second) and iodine load (in grams) delivered over time. The signal from the transmitter was recorded for analysis using a LabVIEW virtual instrument (2012 SPI, National Instruments).

### 2.5. Image Evaluation

For part 1 of the study, time attenuation curves (TAC) were generated (SyngoVia, Siemens Healthineers). For this purpose, a circular region of interest (ROI) was placed in the following target structures: liver (4 ROIs), descending aorta, V. cava, portal vein. To eliminate any residual attenuation from the prior CM application, a baseline correction was performed. In order to determine the optimal contrast phases, the time to peak (TTP) was determined and the TAC for each target structure was analyzed for differences between the protocols.

For part 2 of this study, abdominal parenchymal and vascular target structures were analyzed by a radiologist with more than 10 years of experience in abdominal CT imaging. This was performed by placing a circular ROI in the target structure and, for most structures, performing multiple measurements (indicated in brackets in the following). From the multiple measurements, a mean value was calculated for:

Liver (4 ROIs), pancreas (3 ROI at caput, corpus and cauda), kidney cortex (3 ROI for each side), kidney medulla (3 ROI for each side), descending aorta (1 ROI), V. cava (4 ROI), portal vein (3 ROI), liver vein (3 ROI) renal artery (1 ROI each side), celiac trunk, superior mesenteric artery (1 ROI), splenic artery (1 ROI), renal vein (1 ROI each side), splenic vein (1 ROI), superior mesenteric vein (1 ROI).

A baseline correction was performed for each imaging phase (Phase actual—Phase non-enhanced).

### 2.6. Statistical Analysis

Statistical analysis was performed with SPSS IBM SPSS Statistics, version 20.0 for Macintosh; SPSS, Inc., Chicago, IL, USA). A *p*-value of ≤0.05 was considered statistically significant. Continuous variables were expressed as mean ± standard deviation (SD). A Friedman test (non-parametric one-way repeated-measures analysis) was used to analyze the different injection protocols. Dunn’s multiple comparison tests were performed post-hoc and the results were corrected for multiple comparisons using the Bonferroni method.

## 3. Results

### 3.1. Dynamic CT

**Figure 1:**
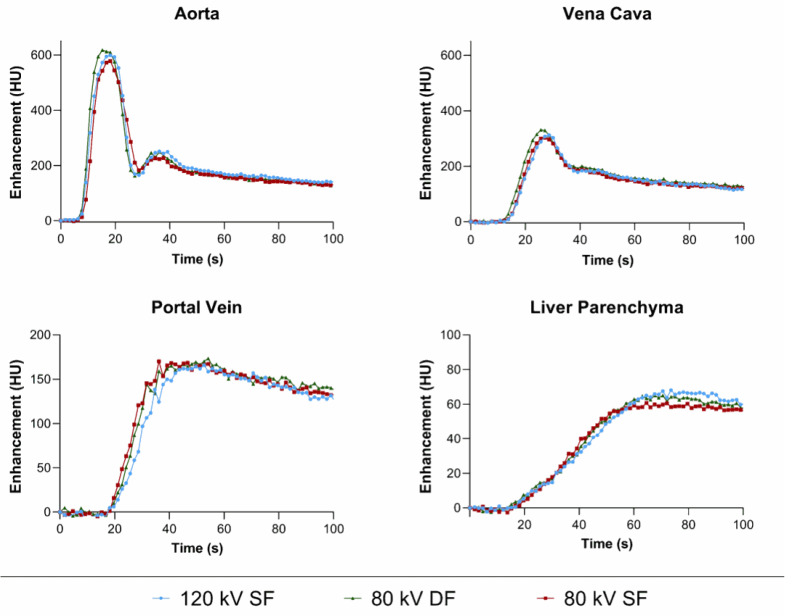
Time attenuation curves for different injection protocols for aorta, vena cava, portal vein, and liver parenchyma. Green curve represents the 80 kV DF protocol; red curve represents the 80 kV SF protocol; blue curve represents the 120 kV SF protocol.

**Figure 2:**
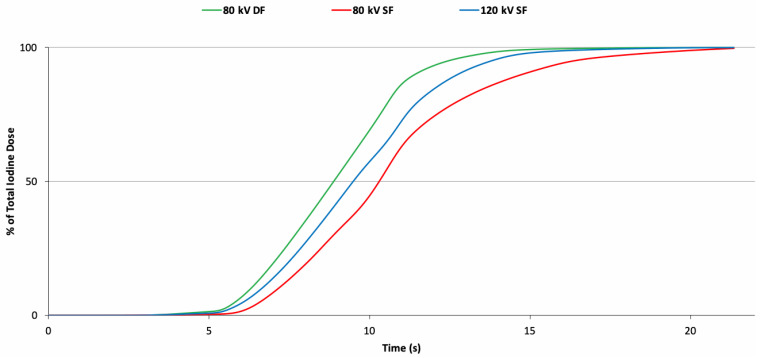
Fluid delivery measurements. Fluid delivery measurements for different injection protocols. Green curve represents the 80 kV DF protocol; red curve represents the 80 kV SF protocol; blue curve represents the 120 kV SF protocol.

In the first part of the experiment, dynamic CT was performed for each protocol. The heart rate for the 80 kV DF (118 ± 14 bpm), 120 kV SF (116 ± 24 bpm), and 80 kV SF (129 ± 11 bpm) examinations did not differ significantly (*p* = 0.3); however, the mean for the 80 kV SF was more than 10% higher than the other two. TACs of the liver, descending aorta, V. cava, and portal vein are presented in Figure 1. Within these ROIs, no obvious differences in the bolus shape (height and width) among injection protocols were observed for any of these structures, i.e., the two 80 kV injection protocols led to almost identical TACs when compared to the 120 kV reference. However, smaller temporal differences were observed particularly in the dynamic phase, where the 80 kV DF protocol resulted in an earlier peak enhancement in the aorta (TTP = 14 ± 5 s) and V. cava (TTP = 26 ± 2 s) compared to the 120 kV reference (TPP = 18 ± 2 s and 28 ± 1 s for aorta and V. cava, respectively). In contrast, the 80 kV SF protocols showed slightly delayed peak enhancement in the aorta (TPP = 20 ± 2 s) but equivalent peak enhancement in the V. cava (28 ± 1 s) when compared to the reference. This result is corroborated with the fluid delivery measurement results shown in Figure 2, demonstrating that the 80 kV DF protocol achieved the delivery of 95% of the total programmed iodine load 25% faster than the 80 kV SF protocol but only 9% faster than the 120 kV SF reference protocol.

**Figure 3:**
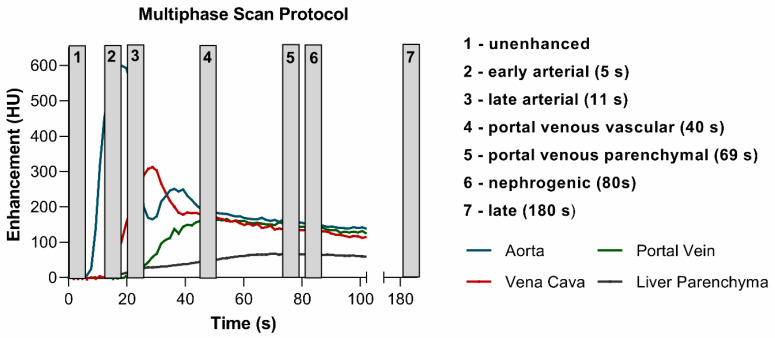
Multiphase scan protocol: Exemplary relative time enhancement curves of aorta, portal vein, V. cava, and the liver parenchyma superimposed with the 7 imaging phases. The start of each phase related to the bolus tracking is given in brackets.

Based on the analysis of dynamic CT exams, the time delays for the different contrast agent phases related to the bolus tracking threshold level were chosen as follows: early arterial 5 s, late arterial 11 s, portal venous vascular 40 s, portal venous parenchymal 69 s, nephrogenic 80 s, late 180 s (Figure 3).

### 3.2. Multiphase Abdominal CT

In the second part of the study, a CT scan of the abdomen was performed at each of the optimized contrast phase timepoints determined in part 1. This resulted in seven CM phases for each injection protocol. The heart rates measured with the 80 kV DF (108 ± 25 bpm), 120 kV SF (115 ± 21 bpm), and 80 kV SF (122 ± 19 bpm) protocols did not differ significantly between groups (*p* = 0.28); however, the mean was once again more than 10% higher for the 80 kV SF compared to the 80 kV DF.

### 3.3. Liver

**Figure 4:**
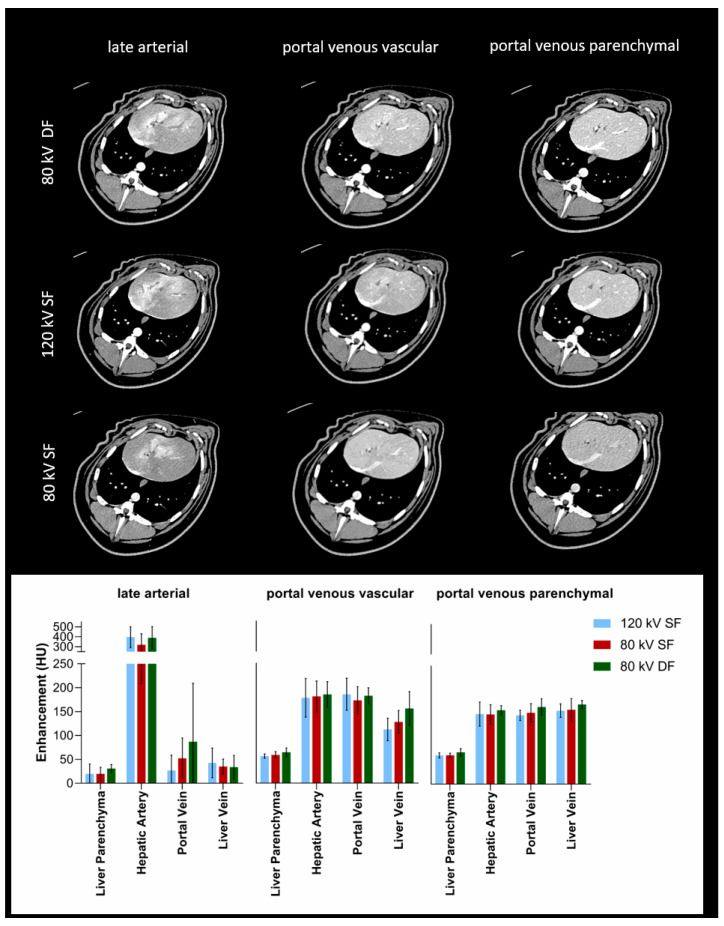
Enhancement of hepatic structures. Columns show the enhancement of clinically relevant structures of the liver for the different injection protocols and clinically relevant phases.

For hepatic structures, no statistically significant differences could be found between the three protocols for the clinically relevant phases (late arterial, portal venous vascular, and portal venous parenchymal; Table 3). Although the differences were not statistically significant, the hepatic artery in the late arterial phase (80 kV DF 390 ± 111 HU; 80 kV SF 319 ± 111 HU; 120 kV SF 396 ± 104 HU) and, to a smaller extent, the portal vein in the portal venous vascular phase (80 kV DF 182 ± 17 HU; 80 kV SF 173 ± 28 HU; 120 kV SF 186 ± 33 HU) showed higher average peak enhancements for the 80 kV DF than for the 80 kV SF protocol and closer equivalence of means to the reference 120 kV SF protocol (Figure 4). In the liver parenchymal phase, the DF protocols (80 kV DF 67 ± 8 HU) also showed a trend of higher enhancement than the other injection protocols (80 kV SF 61 ± 5 HU; 120 kV SF 61 ± 4 HU).

### 3.4. Pancreas

**Figure 5:**
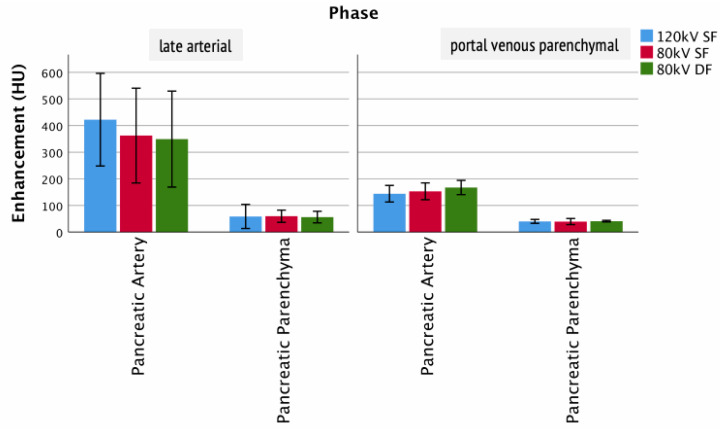
Enhancement of pancreatic structures. Columns show the enhancement of clinically relevant structures of the pancreas for the different injection protocols and clinically relevant phases.

For the pancreas, the presentation of the arterial supply celiac trunk (arterial phase: 80 kV DF 349 ± 145 HU; 80 kV SF 362 ± 143 HU; 120 kV SF 422 ± 140 HU) as well as the pancreatic parenchyma (arterial phase: 80 kV DF 47 ± 5 HU; 80 kV SF 49 ± 4 HU; 120 kV SF 46 ± 8 HU) did not show statistically significant differences (Figure 5). Although the results were not significant, in this ROI, the average peak enhancement for the 80 kV SF was higher than the 80 kV DF, with both lower than the average for the reference 120 kV SF protocol.

### 3.5. Kidney

**Figure 6:**
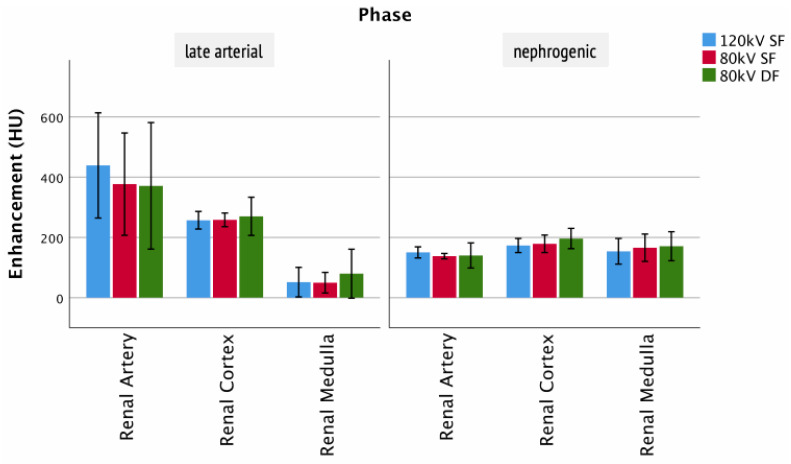
Enhancement of renal structures. Columns show the enhancement of clinically relevant structures of the kidney for the different injection protocols and clinically relevant phases.

For the arterial and nephrogenic phase, the renal cortex and renal medulla as well as the arterial supply showed no significant differences between the injection protocols (Figure 6). Although statistically insignificant, it was observed that in the renal artery in both late arterial and nephrogenic phases, the 120 kV SF protocol led to a higher average peak enhancement than the 80 kV protocols (late arterial: 80 kV DF 371 ± 169 HU; 80 kV SF 377 ± 137 HU; 120 kV SF 439 ± 141 HU/nephrogenic: 80 kV DF 140 ± 26 HU; 80 kV SF 138 ± 7 HU; 120 kV SF 150 ± 15 HU). However, in the renal cortex and the renal medulla, the 80 kV DF protocol trended with higher peak enhancement.

### 3.6. Arteries and Veins

**Figure 7:**
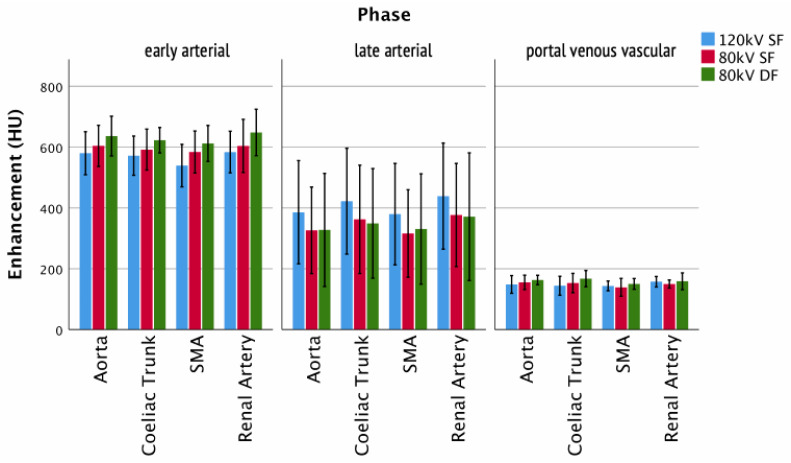
Enhancement of arteries. Columns show the enhancement of arteries for the different injection protocols and clinically relevant phases.

**Figure 8:**
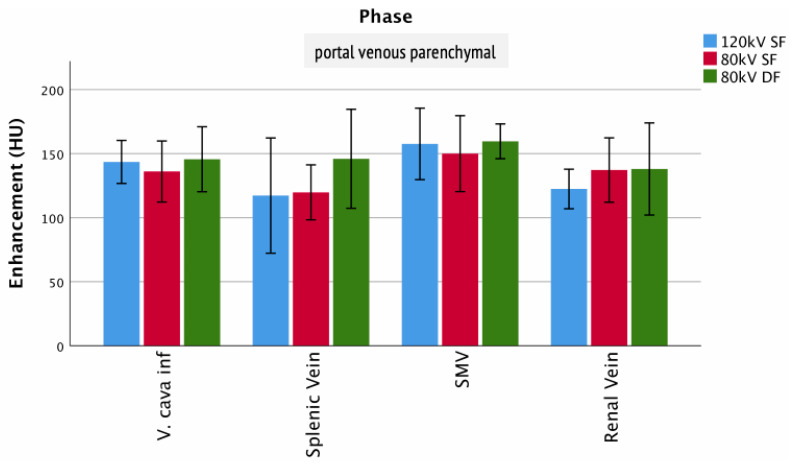
Enhancement of veins. Columns show the enhancement of veins for the different injection protocols in hepatic phase.

The visualization of arteries (early arterial/late arterial/portal venous vascular) showed no significant differences between the protocols (Figure 7), e.g., aortic enhancement (arterial phase: 80 kV DF 328 ± 150 HU; 80 kV SF 326 ± 115 HU; 120 kV SF 386 ± 137 HU). This was equally true for veins in the portal venous vascular CE phase (Figure 8), e.g., V. cava (portal venous vascular phase: 80 kV DF 171 ± 79 HU; 80 kV SF 163 ± 74 HU; 120 kV SF 160 ± 73 HU).

## 4. Discussion

Low kV CT is an established technique in CT angiography that offers the opportunity for reduced radiation exposure as well as a reduction in the CM dose administered [1,2]. However, the potential benefits of low kV CT imaging have not been established for abdominal parenchymal organs. Consequently, this study evaluated the combination of low kV multiphase abdominal imaging (80 kV) systematically in an animal model. Specifically, two adapted low CM dose injection protocols were used for the enhancement of parenchymatous organs and vessels as compared to a standard 120 kV scan and CM protocol. The CM dose was adjusted from the reference 120 kV protocol to the 80 kV protocol based on the literature, including the 10-to-10 rule as well as previous calibration studies [4,10]. The adaptation of the CM injection protocol was carried out using two different techniques. First, a standard reduction in both injection rate and CM volume while keeping injection duration constant was implemented. Second, we maintained the injection rate and total injected volume; however, we administered a contrast–saline mixture of 60%–40%, respectively.

The results of our study allow for the conclusion that the low kV imaging of abdominal parenchymal organs as well as venous and arterial vessels with reduced iodine dose and adapted IDR is feasible in all clinically relevant CE phases. The contrast media (CM) dose used in this study of 300 mgI/kg body weight at 80 kV was found to be sufficient to obtain a liver parenchymal enhancement > 50 HU during the portal venous phase. Both low kV injection protocols, the SF and the DF protocol, lead to peak CT signal enhancements that could not be statistically differentiated from the standard 120 kV reference procedure obtained at full CM dose. Thus, the calibration of the iodine dose to the tube voltage in parenchymal imaging, i.e., the 40% lower dose at 80 kV versus the 120 kV standard, is similar to that obtained in CTA studies [4,13].

Although dynamic CT revealed differences in the TTP analysis for the early vascular phase among the injection protocols, the TACs for these vessels and, in particular, for the liver parenchyma were very similar. The data suggest that the reduced injection rate may not significantly alter the timing of multiphase abdomen imaging. The scan delays between the different contrast phases can be left unchanged when switching from a full CM dose (e.g., 120 kV standard) to adapted low kV injection protocols, as long as the injection duration remains constant by uniformly reducing both the iodine load and IDR using either adaptation method.

A key focal point of this multiphase abdominal study was the visualization of the parenchymatous organs: the liver, pancreas, and kidneys. For these organs, the signal enhancement was in the diagnostic range and no statistically significant differences were found in any of the contrast phases between the individual examination and injection protocols.

At a glance, these results may appear in contrast to the study by Miyoshi et al., who showed a significant difference in quantitative enhancement for liver parenchyma in the arterial, portal venous, and equilibrium phase in 70 kV imaging of the abdomen for reduced iodine load (50%) [14]. Although statistically significant, the maximum difference in the liver enhancement in their study during the portal venous phase was 13 HU (13%), compared to the 6 HU (10%) difference (80 kV DF protocol) in this study. However, taking the relative density differences into account, the results of the two studies are in a similar range. Interestingly, Miyoshi et al. were also able to show that low kV imaging results in better SNR and CNR compared to 120 kV imaging [14]. This is in line with Martens et al., who showed that, for low kV (90 kV) imaging, the CM dose can be adapted to the tube voltage and patient weight without a reduction in image quality [6].

This holds true for the pancreas as well, as no significant differences between the injection protocols could be shown for the parenchyma in any contrast phase. This finding is of particular importance as the evaluation of the pancreas in the clinical setting requires multiphasic protocols [15,16,17]. Both the low kV SF and the DF techniques allow for the parenchyma and the vascular structures to be imaged with comparable CE. Clinical studies by Yoon et al. and Böning et al. show that the detection of hypervascularized lesions in low kV imaging of the liver and pancreas is equivalent to standard kV examination [18,19]. Considering the data of this study, in which the enhancement of the pancreas and liver in both the arterial and portal venous parenchymal phases are similar in low and standard kV imaging for different injection protocols, this study result supports the clinical findings of Yoon et al. and Böning et al.

Another novelty of this study is that each injection protocol equally supported renal cortex and renal medulla visualization in all contrast phases (arterial, nephrogenic, portal venous parenchymal). M. Kanematsu et al. demonstrated improved contrast for both renal vessels and cortex for 80 kV protocols with reduced iodine load [20]. It should be noted, however, that, in this study, only a 33% reduction in iodine dose was achieved between the standard reference 120 kV and 80 kV. In contrast, we demonstrated that a reduction of up to 40% in the iodine dose can be applied between the two kV settings without differences in CE. It is assumed that the non-optimized iodine dose reduction may be the reason for the differences between the results of our study and those of M. Kanematsu et al.

No statistically significant differences were observed between the low kV SF and DF injection protocols. Based on this, both approaches can be used to adapt the iodine dose to the lower tube voltage. The DF approach would allow the use of established injection protocols and adaption of the kV calibration by the contrast to saline ratio. Additionally reflected in the literature and mirrored in the results of part 1 of this study to a smaller degree are differences in the arrival time and bolus shape of the CM comparing the 80 kV DF with the 80 kV SF and 120 kV SF protocols [13]. As shown in the literature with phantom research in which control over simulated cardiac output and blood flow is possible, the DF protocol results in a faster arrival time of the CM into the large thoracic vessels when compared to the reduced injection rate of the adapted SF protocol. The faster arrival time allowed for similar timing to the 120 kV reference protocol [13]. It is postulated that these results were not able to be replicated in this study in a statistically significant way due to variation in the heart rate of the animals. Although the heart rate did not significantly differ among protocols on an intraindividual basis, it was, on average, approximately 10% higher for the 80 kV SF protocol when compared to the 80 kV DF. The associated changes in cardiac output may explain the absence of a significantly higher peak enhancement with DF and the less pronounced differences in timing compared to SF. It is hypothesized that these expected differences between SF and DF protocols may be even more pronounced for CM with a higher iodine concentration and hence viscosities. This is in part because the simultaneous injection of CM and saline in the DF protocol can significantly reduce the fluid viscosity, as viscosity decreases exponentially with iodine concentration [21]. This might be particularly relevant when CM are injected at room temperature, where viscosities may be more than a factor of 2 higher [22]. In this study, we used a standard iodine concentration (300 mgI/mL) that was preheated to 37 °C. Previous CTA studies demonstrated higher vascular attenuation bolus profiles for the 300 mgI/mL formulation compared to very high concentrations (370–400 mgI/mL) [11,23]. The DF dilution may lead to the same effect, which represents an alternative explanation for the trend of higher hepatic attenuation observed for the DF injection protocol in our study.

The results of our study allow the conclusion that low kV abdominal imaging with reduced IDR/iodine dose yields the same contrast as conventional standard 120 kV protocols. In clinical routine, a trade-off is usually made between radiation dose reduction and CM reduction. Especially in younger patient cohorts, the advantage of dose reduction is predominant, whereas in patients with reduced renal function, the possibility of reducing the CM dose plays a more important role [24,25]. In addition, both the DF and SF techniques offer the possibility to adapt the IDR to the ideal kV setting according to the patient’s weight; however, the DF technique is likely to mitigate any negative effects on timing as the injection rate remains constant.

## 5. Study Limitations

The physiology of the spleen in the present animal model does not correspond to the contrast behavior in humans; therefore, these results were not presented. The BMI of the animal model differs from the anticipated BMI in a clinical setting, where higher values would be expected. Our study focuses on the effect of the injection protocol on CE and does not consider CNR and SNR. In fact, the radiation dose was around 20% lower for the 80 kV exams, resulting in a slightly higher noise level than the 120 kV reference images. As a result, an analysis of the SNR and CNR was not performed as the different noise levels may mask the effect of the injection protocols on the CE.

Although initial studies on the detection of parenchymal abdominal organ masses with low kV CT imaging are available, detection with the DF technique has not yet been performed.

Our study was performed on a limited number of animals and the standard deviations of the mean attenuation values were high despite the high degree of standardization. Although the heart rate of the animals was monitored and evaluated, the deviations between animals were higher than would be considered ideal, and this gives only an estimation of other important cardiovascular parameters, such as cardiac output, that influence the timing and distribution of CM throughout the body.

## 6. Conclusions

Multiphase abdominal low kV CT imaging of parenchymal organs and vessels with reduced iodine load and IDR can be adequately performed with adapted CM injection protocols. Both low kV injection techniques, including SF with reduced injection rate and DF with the simultaneous application of CM and saline, yield no significant differences in CE of the liver, pancreas, kidney, and abdominal vessels in comparison to the 120 kV standard CM protocol. This yields the opportunity for patient-individualized contrast media application for diagnostics of parenchymal organs.

## Figures and Tables

**Table 1:** CT scan parameter.

		Study Part 1						
Protocol	Tube Current (mAs)	Collimation (mm)	Rotation Time (s)	Pitch	CDTI (mGy∙cm/s)	Scan Duration (s) and Cycles	Reconstruction	Slice Thickness (mm)
120 kV SF	150	192 × 0.6	0.5	-	10	105/71	Br36	2
80 kV SF	430	192 × 0.6	0.5	-	8.3	105/71	Br36	2
80 kV DF	430	192 × 0.6	0.5	-	8.3	105/71	Br36	2
		**Study Part 2**						
120 kV SF	150	192 × 0.6	0.5	0.85	10	4	Br40/Safire3	1.5
80 kV SF	430	192 × 0.6	0.5	0.85	8.3	4	Br40/Safire3	1.5
80 kV DF	430	192 × 0.6	0.5	0.85	8.3	4	Br40/Safire3	1.5

Listing of the different CT scan parameters for study part 1 and study part 2.

**Table 2:** Injection protocols.

		Contrast Media (CM) Injection (ml/s)		Saline Injection		Dose CM (mgI/kg)
Protocol	Tube Voltage (kV)	Flow Rate CM	DualFlow Saline	Flow Rate Saline (mL/s)	Volume (mL)	
SF 120 kV	120	5	-	5	30	500
SF 80 kV	80	3	-	3	30	300
DF 80 kV	80	3	2	5	30	300

Listing of the different evaluated injection protocols.

**Table 3:** Hepatic peak attenuation.

		Injection Protocol			
Phase	Structure	80 kV DF (HU)	120 kV SF (HU)	80 kV SF (HU)	Friedmann *p*-Value
Late arterial	Liver Parenchyma	30 ± 10	19 ± 21	20 ± 14	0.549
	Hepatic artery	390 ± 111	396 ± 104	319. ± 111	0.247
	Portal vein	87 ± 123	27 ± 32	52 ± 43	0.819
	Liver veins	34 ± 25	41 ± 31	35 ± 16	0.549
Portal venous vascular	Liver Parenchyma	65 ± 9	60 ± 3	59 ± 7	0.247
	Hepatic artery	214 ± 44	178 ± 40	181 ± 32	0.247
	Portal vein	182 ± 17	186 ± 33	173 ± 28	0.549
	Liver veins	156 ± 35	112 ± 24	128 ± 24	0.247
Portal venous parenchymal	Liver Parenchyma	67 ± 8	61 ± 4	61 ± 5	0.549
	Hepatic artery	156 ± 10	148 ± 26	148 ± 20	0.692
	Portal vein	163 ± 17	146 ± 11	151 ± 19	0.247
	Liver veins	169 ± 9	156 ± 14	158 ± 24	0.247

Peak attenuation (mean ± standard deviation) for each scan protocol for relevant hepatic structures and contrast phases. Comparison using Friedmann test. *p* ≤ 0.05 was considered statistically significant.

## Data Availability

The data presented in this study are available within this article.

## Abbreviations

AUC	area under the curve
CM	contrast media
CNR	contrast to noise ratio
CTA	computed tomography angiography
CO	cardiac output
DF	DualFlow
FW300	full width 300
FWHM	full width half max
IDR	iodine delivery rate
kV	kilovoltage
MaxHU	maximum Hounsfield units
SF	single flow
SNR	signal to noise ratio
SV	stroke volume
TTP	time to peak
